# Efficacy and safety of Shenkang injection as adjuvant therapy in patients with diabetic nephropathy: A protocol for systematic review and meta-analysis

**DOI:** 10.1097/MD.0000000000023821

**Published:** 2020-12-24

**Authors:** Yanping Wang, Mingzhu Li, Chenyun Li, Sheng Xu, Jiangfeng Wu, Gaochuan Zhang, Yuanyuan Cai

**Affiliations:** a Evidence-based Medicine Research Center, Jiangxi University of Traditional Chinese Medicine, Jiangxi; b School of Basic Medical Sciences, Guangzhou University of Chinese Medicine, Guangdong, China; c Clinical Medical College of Acupuncture Moxibustion and Rehabilitation, Guangzhou University of Chinese Medicine, Guangdong; d Postgraduate Education; e Institute for Advanced Study, Jiangxi University of Traditional Chinese Medicine, Jiangxi, China.

**Keywords:** Shenkang injection, diabetic nephropathy, protocol, systematic review, meta-analysis

## Abstract

**Background:**

Diabetic nephropathy is a frequent microvascular complication of diabetes mellitus that causes end-stage renal disease most of the time. In China, Shenkang injection is one of widely used traditional Chinese medicine for treating chronic kidney disease, but its efficacy and safety have not yet been clarified. We will systematically review the current randomized controlled trial (RCT) evidence to summarize the efficacy and safety of Shenkang injection in treating diabetic nephropathy.

**Methods:**

We will search 7 literature databases including PubMed, EMBASE, Cochrane Library, Sinomed, Chinese National Knowledge Infrastructure, Wanfang, and VIP. Two trial registry platforms will also be searched. The time frame of the search will be from the inceptions of the databases to December 31, 2020. RCTs assessing Shenkang injection combined with basic treatments versus basic treatments alone for treating diabetic nephropathy will be included. The risk of bias within the individual RCTs will be evaluated using criteria proposed by the Cochrane Handbook 5.1.0. The primary outcomes to be investigated are glomerular filtration rate and serum creatinine; the secondary outcome will include 24-hour urine albumin excretion rate, blood urea nitrogen, fasting blood glucose, postprandial blood glucose, hemoglobin A1c, total cholesterol, triglyceride, response to treatment, and incidence of adverse events. The effect data of individual RCTs by performing random-effects model meta-analysis. Statistical heterogeneity will be measured by the Cochran Q test and *I*-squared statistics. Three subgroup analyses, set based on clinical experience, will be performed to explore the sources of heterogeneity. Sensitivity analyses excluding RCTs with high risk of bias and using fixed effect model will be done to test the robustness of the meta-analytic results. Publication bias across included RCTs will be evaluated by funnel plots and Egger test.

**Results:**

This study will provide systematic review on the efficacy and safety of Shenkang Injection as adjuvant therapy in patients with diabetic nephropathy by rigorous quality assessment and reasonable data synthesis. The results will be submitted to a peer-reviewed journal for publication.

**Conclusion:**

This systematic review will provide the best evidence currently on Shenkang Injection as adjuvant therapy in patients with diabetic nephropathy.

**INPLASY registration number:**

INPLASY2020110014.

## 1 Introduction

### 1.1 Description of the condition

Diabetic nephropathy is a serious microvascular complication caused by diabetes mellitus; it is almost irreversible and will ultimately lead to end-stage renal disease.^[1,2]^ Approximately one-third of patients with type 2 diabetes mellitus will be complicated with diabetic nephropathy.^[3]^ With the increase of prevalence of type 2 diabetes mellitus, the global disease burden of diabetic nephropathy also substantially increased---the global diabetic nephropathy cases increased by 74% and the total disability-adjusted life year caused by diabetic nephropathy increased by 113% from 1990 to 2017.^[4]^

### 1.2 Description of the intervention

Shenkang injection is an injection preparation of Chinese patent medicine that is widely used to treat kidney disease in China.^[5]^ Shenkang injection is composed of 4 herbs, *Rhubarb*, *Salvia miltiorrhiza*, *Radix Astragali*, and *Safflower.* Pharmacological studies have shown that *Rhubarb* and *Radix Astragali* are effective as anti-inflammation and antioxidant agents, *Salvia miltiorrhiza* shows effects on anticoagulation, thrombolysis, and platelet aggregation inhibition, and *Safflower* could vasodilate blood vessels, alleviate damages on vascular endothelium, and reduce blood viscosity.^[6,7]^ The combined use of these herbs could achieve a synergistic effect. The injections of their extracts could improve human bioavailability and accelerate the onset of the treatment effects.^[8]^

### 1.3 How the intervention might work

An experiment on rat with six-fifths nephrectomy model suggested that Shenkang injection could decrease serum creatinine and blood urea nitrogen levels by attenuating glomerular sclerosis and interstitial fibrosis, and reducing inflammatory cell infiltration.^[9]^ At the molecular level, Shenkang injection could induce the increase of levels of anti-aging, gene (e.g., Klotho protein), activation of *Sirt1* gene and peroxisome proliferator activated receptor γ, and inhibition of the expression of mammalian target of rapamycin and *P66shc* gene. The molecular mechanism is associated with resisting aging and oxidative damages on kidney cells caused by hyperglycemia toxicity.^[10]^

### 1.4 Why it is important to perform this review?

The current routine managements for diabetic nephropathy mainly involve the control of the high blood glucose level to delay the progression of kidney injury, and meanwhile treat the frequent comorbidities that affect the kidney such as hypertension and dyslipidemia.^[11,12]^ There is lack of a special approach to repair kidney damages from diabetic nephropathy. Based on the above-mentioned mechanisms and the experiences of clinical uses, we expect that Shenkang injection can be an adjuvant medicine to treat diabetic nephropathy by repairing kidney damages and enhancing renal functions. In fact, multiple randomized controlled trials (RCTs) have been conducted to test the effects of Shenkang injection in patients with diabetic nephropathy, while they are generally small in sample size and difficult to generate a consistent conclusion.

### 1.5 Objective

This systematic review is to clarify the efficacy and safety of Shenkang injection as adjuvant therapy in patients with diabetic nephropathy by summarizing currently available RCT evidence.

## 2 Methods

### 2.1 Study registration

This protocol was registered on the INPLASY platform (registration number: INPLASY2020110014, https://inplasy.com/inplasy-2020-11-0014). We reported the protocol according to the Preferred Reporting Items for Systematic Review and Meta-Analysis Protocols (PRISMA-P) statement. ^[13]^

### 2.2 Inclusion and exclusion criteria

#### 2.2.1 Type of study

Eligible studies will be RCTs, of which randomized cross-over trials will be excluded. For those multiple reports generated from the same study population, the main RCT with longest follow-up period will be retained.

#### 2.2.2 Participants

We will include RCTs that enrolled patients diagnosed with diabetic nephropathy by any recognized diagnostic criteria in which the pathological changes in kidney structure and functions should be caused by type 2 diabetes mellitus (e.g., the American Diabetes Association criteria^[11]^). The patients’ age should be 18 years or elder. There will be no restriction on the stage of kidney damages. We will exclude patients with primary nephropathy or nephropathy secondary to other diseases, such as hypertension, gout, and systemic lupus erythematosus.

#### 2.2.3 Interventions and controls

Shenkang injection is the concerned intervention in this study. We will not limit the dose or course of the treatment, but other preparations with the same herbal compositions as Shenkang injection, such as granules and capsules, will be excluded. We will include RCTs comparing Shenkang injection combined with basic treatments (e.g., antidiabetic treatment, correction of electrolyte disturbances, and control of proteinuria) versus the same basic treatments. Other traditional Chinese medicines will not be allowed in either groups.

#### 2.2.4 Outcomes

##### 2.2.4.1 Primary outcomes

The primary outcomes will be estimated glomerular filtration rate (eGFR, mL/min/1.73 m^2^) and serum creatinine (μmol/L).

##### 2.2.4.2 Secondary outcomes

The following outcomes will also be assessed as the secondary outcomes: two renal function indicators: 24-hour urine albumin excretion rate (g/24 h) and blood urea nitrogen (mmol/L); 3 blood glucose indicators: fasting blood glucose (mmol/L), postprandial blood glucose (mmol/L), hemoglobin a1c (mmol/L); 2 blood lipid indicators: total cholesterol (mmol/L) and triglyceride (mmol/l); 1 qualitative outcome: response to treatment classified by recognized classification criteria, such as guiding principles for clinical research of new Chinese medicines; and 1 safety outcome: incidence of adverse events related to Shenkang injection.

### 2.3 Data sources and Search strategy

Searches will be performed in literature databases of PubMed, EMBASE, Cochrane Library, Sinomed, Chinese National Knowledge Infrastructure, Wanfang, and VIP to collect relevant studies, without language restriction. The time frame of the search will be from the inceptions of the databases up to December 31, 2020. The terms regarding diabetic nephropathy” and “Shenkang injection,” including both free text words and medical subject headings, will be used to construct the search strategy. A search strategy example in PubMed is compiled in Table 1. We will also search 2 trial registration platforms, Clinicaltrials.gov and Chinese Clinical Trial Registry, and the references of relevant reviews to include additional studies.

**Table 1:** Search strategy in PubMed.

No.	Search terms
1	diabetic nephropathy[mh]
2	diabetic nephropathy[tw]
3	diabetic kidney disease[tw]
4	diabetic renal disease[tw]
5	#1 OR #2 OR #3 OR #4
6	shenkang injection[tw]
7	shen kang injection [tw]
8	#6 OR #7
9	animals[mh]
10	humans[mh]
11	#5 AND #8
12	#9 NOT #10
13	#11 NOT #12

### 2.4 Data collection and analysis

#### 2.4.1 Study selection

**Figure 1:**
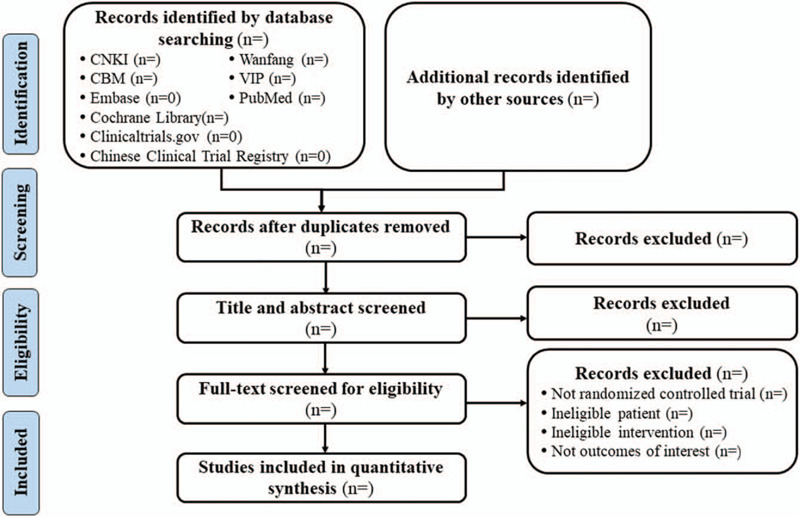
PRISMA-style flow chart of literature screening. CBM = Chinese Biomedical Literature Database; CNKI = China National Knowledge Infrastructure.

The records yielded from the searches will be imported into Endnote X9 (Clarivate Analytics US LLC) for deduplication. Two reviewers working in pairs will independently and repeatedly screen the literatures. They will first exclude irrelevant literatures by reading the titles and abstracts and further go through the full texts to identify the final eligibilities. The reviewers will cross-check the results and address the disagreements in discussions or judgments by a third reviewer. A PRISMA-style flow chart of literature screening, as Figure 1, will be presented.

#### 2.4.2 Data extraction

We will use a standardized, pilot-tested form to extract data of included RCTs. It will be composed of 3 sections: publication characteristics: title, author, journal, and publication time; patients and treatment profiles: gender, age, stage of diabetic nephropathy, complications, dose and course of Shenkang injection, type of control, follow-up period, and proportion and reasons for loss to follow-up; and outcome data: baseline and follow-up measures of each outcome.

#### 2.4.3 Risk of bias assessment

The risk of bias within the included RCTs will be accessed by using risk of bias-assessment tool from the Cochrane handbook 5.1.0 ^[14]^: random number generation method, allocation concealment method, blinding of patients and clinicians, blinding of outcome evaluator, data completeness, selective reporting, and other bias sources. The items 1 and 2 reflect selection bias, while the items 3, 4, 5, and 6 reflect performance bias, detection bias, attrition bias and reporting bias, respectively. Each item will be rated as low, high, or uncertain risk of bias. Two reviewers will independently and repeatedly assess the risk of bias and perform cross-check. Any discrepancy will be addressed in discussions or determined by a third reviewer.

#### 2.4.4 Managing missing data

We will attempt to obtain any missing information from the full text of the RCT by emailing the correspondence authors. Missing standard deviations, if not available from the authors, will be imputed using the median of standard deviations among other RCTs.

#### 2.4.5 Data synthesis

We will pool data extracted from individual RCTs by random effects model meta-analyses in RevMan version 5.3 (Copenhagen: The Nordic Cochrane Centre, The Cochrane Collaboration, 2014). The effects on continuous outcomes will be measured by weighted mean differences (WMDs) or standardized mean difference (SMD) according to their normality, and the inverse variance method will be used as meta-analytic method. The dichotomous outcomes will be measured by risk ratios (RRs) using the Mantel--Haenszel method. The 95% confidence intervals (95% CIs) will be calculated for presenting the estimate precision.

#### 2.4.6 Assessment of heterogeneity

In the meta-analyses, we will use the Cochran Q test to determine whether there is statistically significant statistical heterogeneity among the RCTs, with a calculation of *I*-squared statistics to quantitatively detect the level of heterogeneity. A significant heterogeneity will be defined by a *P* value lower than 0.10 in the Cochran Q test or an *I*^2^ higher than 50%; and in that case, we will further perform subgroup analyses to explore the source of heterogeneity.

#### 2.4.7 Subgroup analysis

Based on the empirical prediction from clinical applications of Shenkang injection, we will perform the following subgroups analyses to explore the sources of heterogeneity:

(1) subgroups analyses stratified by the stage of diabetic nephropathy: we will compare RCTs enrolling patients with stages 1 to stage 3 of diabetic nephropathy with those enrolling patients with stage 4 to stage 5 of diabetic nephropathy; the former patients are expected to have better efficacy.
(2) subgroups analyses stratified by daily dose of Shenkang injection: we will compare RCTs in which the dose of Shenkang injection is >60 mL/day with those ≤ 60 mL/day; the former dose is expected to have better efficacy.
(3) subgroups analyses stratified by course of treatment: we will compare RCTs with a course of treatment >14 days versus those ≤14 days; the former dose is expected to have better efficacy.

#### 2.4.8 Sensitivity analysis

In order to validate the robustness of the meta-analytic estimates, we will perform the following 2 sets of sensitivity analyses: excluding studies with a high risk of bias; and using fixed effects model to pool the data in the meta-analyses.

#### 2.4.9 Assessment of publication bias

We will draw funnel plots and do Egger test to determine whether there is a significant publication bias for any outcomes where at least RCTs are included.

#### 2.4.10 Assessment of the quality of evidence

We will assess the quality of evidence for the results of each outcome based on the Grading of Recommendations Assessment, Development and Evaluation (GRADE) framework. All results will start from high-quality evidence, but the quality of evidence will be rated down according to 5 aspects of limitations, including risk of bias, indirectness, inconsistency, imprecision, and publication bias. The quality of evidence will be finally determined to be high (no obvious limitations for all aspects), moderate (rated down for 1 grade), low quality (rated down for 2 grades), or very low quality (rated down for 3 grades or more).

### 2.5 Ethics and dissemination

This study does not involve personal and human test data, trial data, and therefore does not require ethical approval. We aim to publish the results of this systematic review in a peer-reviewed journal.

## 3 Discussion

A previous systematic review has assessed the effects of Shenkang injection on chronic kidney diseases ^[15]^; however, there was a significant patient-level heterogeneity in its meta-analyses because it included all patients with all types of chronic kidney diseases. In addition, the previous review only focused on patients’ coagulation function but did not assess the outcomes regarding kidney function, such as eGFR and serum creatinine. In contrast to the previous review, we will only focus on diabetic nephropathy, the most common indication in the clinical application of Shenkang injection, and will mainly assess the effects on kidney functions. This will help to reduce heterogeneity and specifically optimize the treatment regimens.

In this systematic review, we will do every effort to enhance reliability of results, including but not limited to systematical literature search, rigorous risk of bias, reasonable data analysis, objective quality of evidence assessment, and conducting the research strictly according to the protocol. We believe the findings of this systematic review will provide the current best available evidence currently on the efficacy and safety of Shenkang injection in the adjuvant treatment of diabetic nephropathy.

## Author contributions

**Conceptualization:** Gaochuan Zhang, Yuanyuan Cai

**Funding acquisition:** Gaochuan Zhang

**Investigation:** Chenyun Li, Sheng Xu, Jiangfeng Wu

**Methodology:** Yanping Wang, Mingzhu Li

**Writing – original draf**t: Yanping Wang, Mingzhu Li

**Writing – review & editing:** Gaochuan Zhang, Yuanyuan Cai
